# Develop a Novel Signature to Predict the Survival and Affect the Immune Microenvironment of Osteosarcoma Patients: Anoikis-Related Genes

**DOI:** 10.1155/2024/6595252

**Published:** 2024-03-27

**Authors:** Mingyi Yang, Yani Su, Ke Xu, Haishi Zheng, Yongsong Cai, Pengfei Wen, Zhi Yang, Lin Liu, Peng Xu

**Affiliations:** Department of Joint Surgery, HongHui Hospital, Xi'an Jiaotong University, Xi'an, Shaanxi, China

## Abstract

**Objective:**

Osteosarcoma (OS) represents a prevalent primary bone neoplasm predominantly affecting the pediatric and adolescent populations, presenting a considerable challenge to human health. The objective of this investigation is to develop a prognostic model centered on anoikis-related genes (ARGs), with the aim of accurately forecasting the survival outcomes of individuals diagnosed with OS and offering insights into modulating the immune microenvironment.

**Methods:**

The study's training cohort comprised 86 OS patients sourced from The Cancer Genome Atlas database, while the validation cohort consisted of 53 OS patients extracted from the Gene Expression Omnibus database. Differential analysis utilized the GSE33382 dataset, encompassing three normal samples and 84 OS samples. Subsequently, the study executed gene ontology and Kyoto encyclopedia of genes and genomes enrichment analyses. Identification of differentially expressed ARGs associated with OS prognosis was carried out through univariate COX regression analysis, followed by LASSO regression analysis to mitigate overfitting risks and construct a robust prognostic model. Model accuracy was assessed via risk curves, survival curves, receiver operating characteristic curves, independent prognostic analysis, principal component analysis, and t-distributed stochastic neighbor embedding (t-SNE) analysis. Additionally, a nomogram model was devised, exhibiting promising potential in predicting OS patient prognosis. Further investigations incorporated gene set enrichment analysis to delineate active pathways in high- and low-risk groups. Furthermore, the impact of the risk prognostic model on the immune microenvironment of OS was evaluated through tumor microenvironment analysis, single-sample gene set enrichment analysis (ssGSEA), and immune infiltration cell correlation analysis. Drug sensitivity analysis was conducted to identify potentially effective drugs for OS treatment. Ultimately, the verification of the implicated ARGs in the model construction was conducted through the utilization of real-time quantitative polymerase chain reaction (RT-qPCR).

**Results:**

The ARGs risk prognostic model was developed, comprising seven high-risk ARGs (CBS, MYC, MMP3, CD36, SCD, COL13A1, and HSP90B1) and four low-risk ARGs (VASH1, TNFRSF1A, PIP5K1C, and CTNNBIP1). This prognostic model demonstrates a robust capability in predicting overall survival among patients. Analysis of immune correlations revealed that the high-risk group exhibited lower immune scores compared to the low-risk group within our prognostic model. Specifically, CD8+ T cells, neutrophils, and tumor-infiltrating lymphocytes were notably downregulated in the high-risk group, alongside significant downregulation of checkpoint and T cell coinhibition mechanisms. Additionally, three immune checkpoint-related genes (CD200R1, HAVCR2, and LAIR1) displayed significant differences between the high- and low-risk groups. The utilization of a nomogram model demonstrated significant efficacy in prognosticating the outcomes of OS patients. Furthermore, tumor metastasis emerged as an independent prognostic factor, suggesting a potential association between ARGs and OS metastasis. Notably, our study identified eight drugs—Bortezomib, Midostaurin, CHIR.99021, JNK.Inhibitor.VIII, Lenalidomide, Sunitinib, GDC0941, and GW.441756—as exhibiting sensitivity toward OS. The RT-qPCR findings indicate diminished expression levels of CBS, MYC, MMP3, and PIP5K1C within the context of OS. Conversely, elevated expression levels were observed for CD36, SCD, COL13A1, HSP90B1, VASH1, and CTNNBIP1 in OS.

**Conclusion:**

The outcomes of this investigation present an opportunity to predict the survival outcomes among individuals diagnosed with OS. Furthermore, these findings hold promise for progressing research endeavors focused on prognostic evaluation and therapeutic interventions pertaining to this particular ailment.

## 1. Introduction

Osteosarcoma (OS) constitutes a malignant neoplasm originating from mesenchymal cells, giving rise to malignant spindle stromal cells that mimic bone tissue [1]. These tumor cells exhibit distinctive characteristics, capable of generating immature osteoid cells. Early clinical manifestations of OS commonly include pain and the presence of a localized mass, contributing to the heightened challenge of accurately diagnosing or recognizing OS during its initial phases. Positioned as the predominant primary bone tumor among adolescents, OS ranks as the third most prevalent cancer, following lymphoma and brain cancer [2]. Incidence primarily affects children and adolescents, yet noteworthy occurrences also arise in individuals above 60 years of age. The global incidence of OS stands at approximately 1–3 cases per million individuals annually [3]. Notably aggressive local invasion and early metastasis serve as primary hallmarks of OS malignancy. The lung represents the most frequent site of metastasis, with a majority of OS patients developing lung metastases within 1 year postdiagnosis, subsequently leading to a dismal prognosis [1]. The prevailing standard of clinical intervention for OS involves extensive surgical resection, coupled with neoadjuvant and adjuvant chemotherapy [4]. Presently, adjuvant chemotherapy post-tumor resection exhibits efficacy in managing OS patients. Despite advancements, this therapeutic approach enhances the 10-year survival rate to roughly 50%; however, patients with metastatic OS continue to endure low survival rates [2]. Substantial progress in elevating survival rates for OS patients remains elusive. Hence, further comprehensive investigations into OS are imperative to enhance therapeutic effectiveness and overall survival rates.

Cells undergo growth and differentiation under physiological conditions, whereas apoptosis is triggered in response to aberrant circumstances. The spatial positioning of cells within tissues necessitates specific mediators, such as the extracellular matrix (ECM), and precise interactions with neighboring cells [5]. The equilibrium of normal tissue relies on the essential processes of cell proliferation and apoptosis. Notably, in the absence of ECM, typically adherent cells undergo a distinct form of apoptotic demise known as anoikis [6]. Integrin, despite lacking a kinase domain, serves as a pivotal factor in mediating ECM adhesion. It interfaces with other molecules to transmit signaling cues, thereby regulating fundamental cellular functions encompassing proliferation, survival, and migration [6]. Anoikis, as a specialized form of apoptotic cell death, is triggered by the disruption of cell/ECM interactions, presenting unique signals that instigate cell demise distinct from other proapoptotic stimuli. This process unfolds in response to extensive loss of cell adhesion, deploying diverse signaling and apoptotic pathways, but is not a singular inducer of cell death [5]. Specifically, anoikis occurs due to inadequate or inappropriate cell–matrix interactions, contributing significantly not only to tissue equilibrium and development but also to carcinogenic processes [7]. It represents a programed cell death mechanism that materializes when cells are separated from the appropriate ECM, thereby impeding integrin connections and preventing cellular dysplasia or attachment to unsuitable substrates [8]. Anoikis assumes a pivotal role in maintaining tissue homeostasis and development by forestalling isolated epithelial cells from establishing colonies elsewhere. Moreover, its dysregulation in certain diseases underscores its involvement in the physiological processes governing tissue equilibrium and development [8].

Anoikis functions as a barrier to cellular metastasis, a phenomenon precipitated by the detachment of cells from the ECM or neighboring cellular entities. Tumor cells harboring malignant propensities have evolved intricate antioxidant mechanisms to endure detachment from their original site and traverse through the lymphatic and circulatory systems. Anomalies within the caspase-activated death receptor pathway, such as the overexpression of FLIP, can engender cellular resistance to anoikis [9]. Consequently, cells resilient to anoikis can circumvent signals triggering death due to detachment, thereby prolonging their survival during migration to secondary sites. Consequently, the resistance to anoikis has been recognized as a pivotal step in the genesis of tumors [10], fostering prolonged survival of detached cells and contributing significantly to cellular immigration and regeneration. Anomalous regulation of anoikis has been designated as a hallmark of malignant transformation, leading to the development of resistance to anoikis and consequent pathogenesis [11]. This underscores a close association between anomalous regulation of anoikis, early metastasis, and overall survival, suggesting anoikis as a pivotal barrier to cellular metastasis. Empirical evidence indicates the contributory role of Src activity in conferring tolerance to anoikis in human SAOS-2 cells. Overexpression of Src has been identified in anoikis-resistant SAOS cells, and the pharmacological inhibition of its activity has demonstrated the restoration of sensitivity to anoikis [11]. The collaborative action of the RanBP9/TSSC3 complex has been revealed to suppress anoikis resistance and metastasis in OS by impeding Src-mediated Akt signaling [12]. Notably, human OS cells exhibiting resistance to anoikis exhibit pronounced angiogenesis by activating the MAPK pathway mediated by Src kinase [13]. Furthermore, anoikis resistance mediated by FASN has been found to promote the growth and metastasis of OS [14]. Metastasis, a multifaceted biological process inherent in most solid tumors, stands as the primary cause of cancer-associated mortality. Consequently, therapeutic interventions aimed at impeding metastasis may serve as compensatory strategies in the battle against cancer.

The utilization of risk prognostic models holds considerable promise in the accurate anticipation of tumor prognosis. These novel models are increasingly employed to forecast the prognostic outcomes among patients affected by diverse tumor types. Specifically, within pediatric populations, a hypoxia gene-based signature has exhibited the capability to predict survival rates while exerting influence on the tumor's immune microenvironment [15]. Moreover, the development of an autophagy-related clinical prognosis model has been instrumental in forecasting overall survival in OS cases [16]. Notably, in the context of OS, a recent approach involving a novel pyroptosis-related signature has proven effective in prognostication and the identification of immune microenvironment characteristics [17]. Another recent investigation has successfully developed a cuproptosis-related long non-coding RNA (lncRNA) risk prognostic model tailored to guide prognosis and assess the immune microenvironment among OS patients [18]. Presently, our study contributes by constructing a pioneering risk prognostic model based on anoikis-related genes (ARGs) to delineate the survival prospects and immune microenvironmental in OS patients. This newly devised prognostic model exhibits efficacy in predicting survival outcomes for individuals with OS.

## 2. Materials and Methods

### 2.1. Data Acquisition and Collation

The study employed data from two distinct cohorts to explore the transcriptomic landscape in OS. The primary cohort consisted of 86 OS cases sourced from The Cancer Genome Atlas (TCGA) database (https://portal.gdc.cancer.gov/). This dataset encompassed comprehensive clinical information, including gender, age, metastatic status, primary tumor site, and specific tumor location. For independent validation, the GSE21257 dataset from the Gene Expression Omnibus (GEO) database (https://www.ncbi.nlm.nih.gov/geo/) was utilized, comprising 53 OS cases with corresponding clinical attributes such as gender, age, metastatic status, and tumor location. Moreover, differential analysis was conducted utilizing the GSE33382 dataset from the GEO database, which incorporated three normal tissue samples and 84 OS tissue samples. This dataset facilitated the exploration of alterations in gene expression between normal and OS tissues. A selection of 794 ARGs was made based on curated information from GeneCards (https://www.genecards.org/) and corroborated by existing literature [19]. Analysis of these datasets enabled the identification of differentially expressed ARGs in OS tissues compared to normal tissues, providing insight into their putative involvement in the pathogenesis of OS.

### 2.2. OS-Related Differentially Expressed ARGs

Initially, it is imperative to ascertain the quantity of ARGs inherent in the genomic constituents of the OS transcriptome dataset. To ascertain the presence of OS-related ARGs, a comprehensive set comprising 794 ARGs was cross-referenced with the genes within the OS transcriptome dataset. Subsequently, an examination is required to elucidate the differential expression patterns of OS-related ARGs within the OS context. Differential expression analysis was conducted on the GSE33382 dataset utilizing the limma package available in the R programing environment. The screening methodology used a significance threshold of *P*  < 0.05 and |logFC| ≥ 0.5, aimed at identifying differentially expressed genes (DEGs) of OS [20]. The genes resulting from the intersection of OS-related ARGs with the OS's DEGs are denoted as OS-related differentially expressed ARGs.

### 2.3. Enrichment Analysis

To elucidate the biological functions associated with OS-related differentially expressed ARGs, a comprehensive roster of genes was submitted for analysis to the Database for Annotation, Visualization, and Integrated Discovery (DAVID, http://david.abcc.ncifcrf.gov/). This facilitated a rigorous functional enrichment assessment utilizing gene ontology (GO) and the Kyoto encyclopedia of genes and genomes (KEGG) [21]. The GO enrichment analysis encompassed three principal categories—biological process (BP), cellular component (CC), and molecular function (MF)—serving as metrics to ascertain the functional implications of the OS-related differentially expressed ARGs. The screening criterion was *P*  < 0.05.

### 2.4. Construction of Risk Prognostic Model

The study utilized univariate COX regression analysis within the survival package of R to discern differentially expressed ARGs significantly associated with OS prognosis (*P*  < 0.01). To mitigate overfitting risks and ascertain the optimal number of ARGs for model construction, LASSO regression analysis employing the glmnet package in R was additionally employed [18, 22]. Identified differentially expressed ARGs linked to OS prognosis via the LASSO regression model underwent validation within an independent cohort (GSE21257). Subsequently, only validated genes were selected for further analysis [16]. Following the identification and validation of differentially expressed ARGs associated with OS prognosis, distinct risk prognosis models were developed for both the training and validation cohorts. These models were leveraged to compute a riskScore based on ARG expression levels for each patient. Higher riskScores corresponded to an elevated likelihood of mortality, allowing for the prediction of individual patient survival probabilities. Calculate riskScore:

(1) $$\begin{array}{c} \text{RiskScore}=\sum_{i=1}^{n}\left(\text{mrna}{\text{exp}}_{i}\times {\text{coef}}_{i}\right). \end{array}$$

The variable “*n*” represents the total number of differentially expressed ARGs that have been found to be associated with OS prognosis. “*i*” represents the *i*th differentially expressed ARG that is associated with OS prognosis. The regression coefficient, denoted by “coef,” is a measure of the strength and direction of the association between the expression level of the differentially expressed ARG and OS prognosis. In the computation of the sample riskscore for individual patients, the methodology involves the multiplication of the expression levels pertaining to each differentially expressed ARGs associated with OS prognosis by their respective regression coefficients. The resultant products are subsequently aggregated, yielding the riskScore for each OS sample [17, 18]. The OS transcriptome under consideration encompasses a total of 86 samples. Following the acquisition of individual riskScore for each OS sample, a sorting procedure based on the riskScore was implemented, resulting in the division of the 86 OS patients into distinct high-risk and low-risk groups, demarcated by the median value of the riskScore.

### 2.5. Validation of the Risk Prognostic Model

The statistical programing language R was employed for conducting diverse analyses aimed at investigating the intricate relationship between risk prognosis, survival outcomes, and differentially expressed ARGs. Initially, various visual representations including risk curves, survival status maps, and risk heatmaps were generated using R for both the training and validation cohorts. These visualizations facilitated the observation of differences in survival time and the identification of differentially expressed ARGs associated with overall survival prognosis among distinct risk groups [16]. Furthermore, the survival and survminer packages within R were utilized to construct survival curves for both cohorts, allowing for an exploration into potential variations in patient survival based on cohort types in the context of OS. Additionally, the timeROC package in R was employed to plot receiver operating characteristic (ROC) curves for the training and validation cohorts, enabling the observation of 1-, 3-, and 5-year survival rates among OS patients involved in the risk prognosis model construction. Finally, the survival package in the *R* was utilized to perform independent prognostic evaluations employing both univariate and multivariate COX regression analyses, separately for the training and validation cohorts [15, 18]. Substantiation of the significance of riskScore in both univariate and multifactorial independent prognostic analyses (*P*  < 0.05) underscores its potential utility as an autonomous prognostic determinant for patients with OS. These analyses aimed to evaluate the potential viability of riskScore and clinical features as independent prognostic factors in OS.

### 2.6. Principal Component Analysis (PCA) and t-Distributed Stochastic Neighbor Embedding (t-SNE) Analysis

PCA and t-SNE analyses were conducted on the risk prognostic model developed from distinct cohorts: the training cohort and the validation cohort, respectively. The primary aim was to ascertain the capacity of the expression patterns exhibited by differentially expressed ARGs, and integrated within the model, in effectively segregating patients into high- and low-risk groups. These analyses served as evaluative measures to test the fidelity and predictive accuracy of the constructed model [18].

### 2.7. Establish and Evaluate ARGs-Clinical Nomogram in the Training Cohort

The nomogram represents a pivotal methodology employed in forecasting cancer prognosis, enabling clinicians to gauge survival probabilities based on distinct clinical characteristics of patients. This investigation sought to construct a nomogram aimed at prognosticating survival rates in individuals diagnosed with OS, leveraging pertinent clinical attributes such as gender (male, female), metastatic status (metastatic, nonmetastatic), primary tumor site (upper limb, lower limb + pelvis), and specific tumor location (upper limb, lower limb + pelvis). The development of predictive nomograms utilizing the RMS package in R facilitated the projection of survival probabilities for OS patients across intervals of 1, 3, and 5 years [17]. Furthermore, calibration curve plotting was employed to assess the concordance between predicted and observed probabilities, thereby validating the accuracy of the nomogram's predictions. This rigorous analysis furnishes clinicians with essential insights for predicting cancer prognosis with greater precision and reliability.

### 2.8. Gene Set Enrichment Analysis (GSEA)

In the analysis of our training cohort, we conducted GSEA to discern the distinct activation pathways within the high- and low-risk groups. Employing specialized GSEA software facilitated the comparison of pathways within predefined gene sets, across these distinct sample groups. Furthermore, to enhance the clarity and visual representation of the pathway enrichment outcomes, we utilized the ggplot2 package in the R programing environment.

### 2.9. Tumor Microenvironment Analysis

The examination of the tumor microenvironment plays a pivotal role in elucidating the underlying biological mechanisms steering cancer progression and response to treatment. This research endeavors to scrutinize the tumor microenvironment among patients with OS utilizing transcriptome data. The investigation leverages the limma and estimation packages of R software to derive stromal scores, immune scores, and total scores for individual patients [22]. Specifically, the stromal score delineates the relative abundance of stromal cells within the tumor microenvironment, while the immune score signifies the extent of infiltration by immune cells. The composite total score amalgamates both stromal and immune scores, serving as an aggregate representation of the overall tumor microenvironmental state. Our inquiry delves into discerning significant disparities in stromal scores, immune scores, and total scores between high- and low-risk groups within the training cohort. To this end, statistical analyses were conducted employing the limma and ggpubr packages within the R environment [17, 18].

### 2.10. Single-Sample Gene Set Enrichment Analysis (ssGSEA)

To elucidate the immune cell composition and functional aspects within OS patients, an analysis employing ssGSEA on transcriptomic data was conducted. This investigation involved the utilization of three distinct R packages: GSVA, limma, and GSEABase. Through the application of these computational tools, enrichment scores for diverse immune cell types and functions pertaining to the immune system were generated. Subsequent comparative analysis was performed between high- and low-risk groups within the training cohort to discern variations in both immune cell composition and functional attributes. This comparative evaluation was executed employing R packages such as limma, reshape2, and ggpubr, enabling effective comparison between the two groups [17, 18].

### 2.11. Immune Infiltration Cell Correlation Analysis

The transcriptomic profiles of 22 infiltrating immune cells in OS were assessed utilizing the CIBERSORT software implemented through R, specifically employing e1071, parallel, and preprocessCore packages [23]. Samples exhibiting a *P*  > 0.05 in the infiltrating immune cell data were systematically excluded from further analysis. To elucidate the association between these 22 infiltrating immune cells and the OS prognosis differentially expressed ARGs of training cohort, correlation analyses were performed using the limma, reshape2, and ggpubr packages in R. Additionally, correlation assessments were conducted between the 22 immune cell types and the riskScore of training cohort, employing the limma, ggplot2, and ggpubr packages.

### 2.12. Correlation Analysis between Clinical Features, ImmuneScore, and RiskScore

The statistical programing environment R was employed to conduct correlation analysis among clinical features, immuneScores, and riskScores within the training cohort's risk prognostic model. Specifically, the analytical procedures utilized functionalities from the limma and ggpubr packages within R. Furthermore, the investigation sought to ascertain notable variations in risk stratification across distinct clinical cohorts as well as between patient groups categorized by high and low immuneScores. These findings will provide valuable insights into the potential clinical significance of the immuneScore and riskScore in predicting patient outcomes.

### 2.13. Differential Analysis of Immune Checkpoints

In order to examine variations among immune checkpoint-related genes within risk prognostic models of the training cohort, the statistical software R was utilized, employing the limma, reshape2, ggplot2, and ggpubr packages. The primary objective of this investigation was to discern the differential expression patterns of immune checkpoint-related genes between high- and low-risk groups [15]. The results of this analysis provide insights into the potential role of immune checkpoint-related genes in predicting the prognosis of the training cohort and may inform the development of novel prognostic markers and therapeutic strategies for managing high-risk patients.

### 2.14. Drug Sensitivity Analysis

In this investigation, we conducted an assessment of drug sensitivity concerning the risk prognostic model within the training cohort. Utilizing analytical tools including the limma, ggpubr, and pRRophetic packages within the *R* environment. Specifically, a screening criterion of statistical significance at *P*  < 0.001 was employed to delineate drugs exhibiting notable sensitivity disparities [18]. The ultimate goal of this analysis was to identify potential therapeutic agents that could improve OS outcomes.

### 2.15. Cell Culture

The human OS cell lines, namely 143B, and U2OS, along with the human normal osteoblast cell line (hFOB1.19), were procured from Wuhan Procell Life Science and Technology Co., Ltd. (Wuhan, China). Cultivation of each cell line was conducted utilizing the corresponding specialized medium obtained from Wuhan Procell Life Science and Technology Co., Ltd., Wuhan, China. The human OS cell lines were maintained under standard incubation conditions at 37°C in an atmosphere enriched with 5% CO_2_. The hFOB1.19 cells were cultured in a distinct incubator set at 34°C with 5% CO_2_.

### 2.16. Real-Time Quantitative Polymerase Chain Reaction (RT-qPCR)

Total RNA extraction was performed on OS cell lines and human fetal osteoblast cell line hFOB1.19 using TRIzol Reagent (Cat. No. P118-05, GenStar, Beijing, China) in accordance with the manufacturer's provided protocols. Subsequently, total RNA underwent amplification through RT-qPCR utilizing SYBR Green Master Mix (Cat#: C0006, TOPSCIENCE, Shanghai, China) following the manufacturer's guidelines, facilitating the quantification of mRNA levels related to ARGs. Primer pairs specific to the target genes were synthesized by Accurate Biology (Changsha, China). Standardization to GAPDH was employed for all samples, and the 2^−*ΔΔ*Ct^ method was applied to assess the relative expression levels.

### 2.17. Statistical Analysis

The present study conducted statistical analyses and visualizations employing the R software v4.1.2, GraphPad Prism v8.2.1, and SPSS 22.0. R software is acknowledged for its extensive suite of tools and libraries specifically designed for diverse data analysis tasks, encompassing hypothesis testing, regression analysis, and data visualization. It is noteworthy that a significance level of *P*  < 0.05 was adopted in this investigation to establish statistical significance in the bioinformatics analysis, in accordance with the widely accepted standard prevalent across various research domains. The presentation of experimental results adhered to the convention of expressing values as mean ± SD (standard deviation), with statistical significance determined through one-way ANOVA. A significance threshold of *P*  < 0.05 was deemed indicative of statistical significance. Furthermore, each experiment was conducted independently at least three times.

## 3. Results

### 3.1. OS-Related Differentially Expressed ARGs

**Figure 1:**
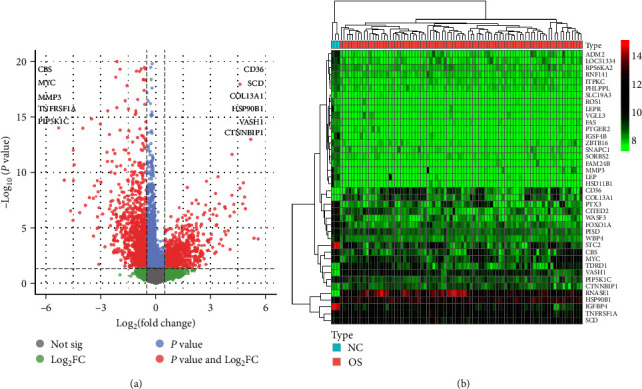
The differential analysis of the GSE33382 dataset. (a) Volcano plot of differentially expressed genes, the left side is low expression and the right side is high expression. (b) Heatmap of differentially expressed genes, with high expression in red and low expression in green.

**Figure 2:**
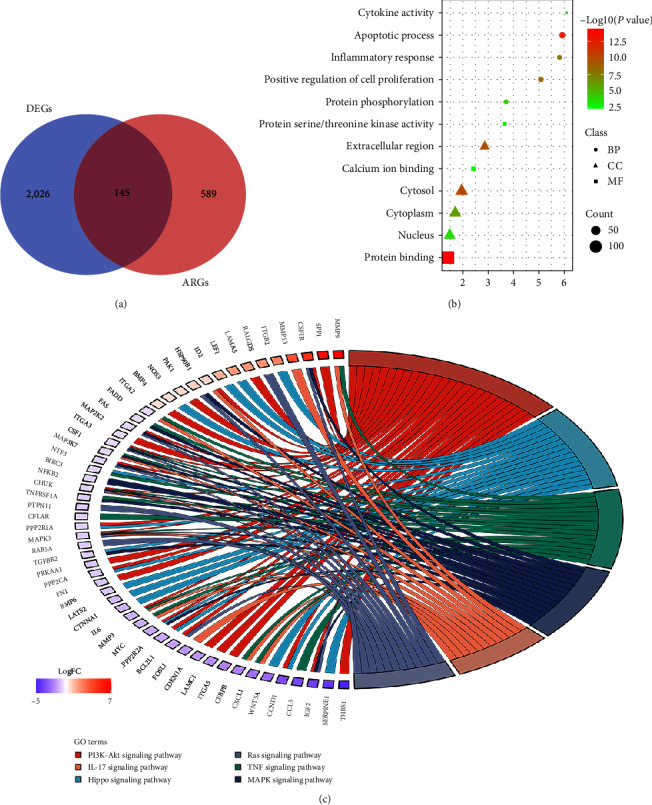
Differentially anoikis-related genes. (a) 145 OS-related differentially expressed anoikis-related genes. (b) Gene ontology enrichment analysis. (c) Kyoto encyclopedia of genes and genomes enrichment analysis.

We intersected 794 ARGs and genes in the OS transcriptome data and obtained 734 OS-related ARGs. Concurrently, a differential analysis of the GSE33382 dataset revealed 2,171 DEGs, encompassing 902 upregulated and 1,269 downregulated genes. Visual representation using R generated volcano plots (Figure 1(a)) and heatmaps (Figure 1(b)) to illustrate these differential expressions. Subsequent intersection of the 2,171 DEGs with the 734 OS-related ARGs resulted in the identification of 145 OS-related differentially expressed ARGs (Figure 2(a)).

### 3.2. Enrichment Analysis

GO and KEGG enrichment analysis were performed on 145 OS-related differentially expressed ARGs. The 145 OS-related differentially expressed ARGs are significantly enriched in apoptotic process, positive regulation of cell proliferation, inflammatory response, and protein phosphorylation in BP. The 145 OS-related differentially expressed ARGs are significantly enriched in cytosol, cytoplasm, nucleus, and extracellular region in CC. The 145 OS-related differentially expressed ARGs are significantly enriched in protein binding, calcium ion binding, protein serine/threonine kinase activity, and cytokine activity in MF. In pathway enrichment, 145 OS-related differentially expressed ARGs are significantly enriched in PI3K–Akt signaling pathway, Hippo signaling pathway, TNF signaling pathway, MAPK signaling pathway, IL-17 signaling pathway, and Ras signaling pathway (Figures 2(b) and 2(c)).

### 3.3. Construction of Risk Prognostic Model

**Figure 3:**
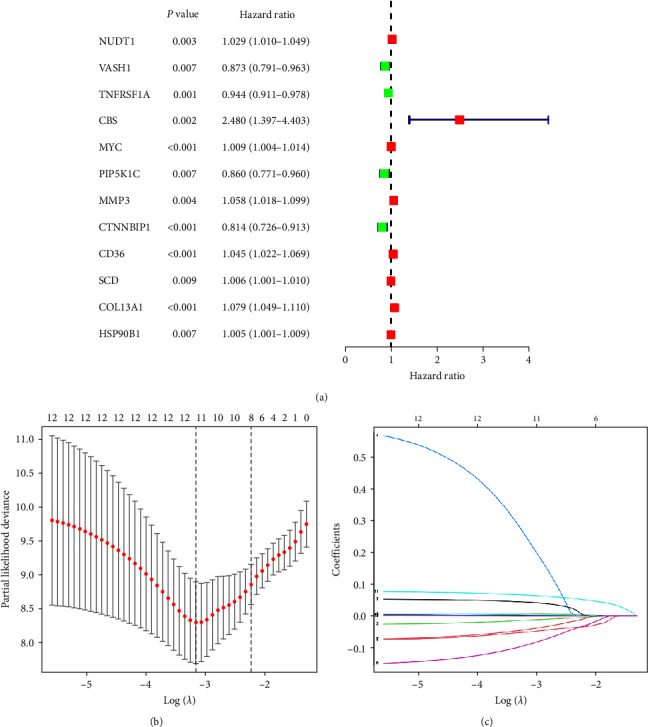
Construction of risk prognostic model. (a) Univariate Cox regression analysis obtained 12 candidate prognostic anoikis-related genes for OS. (b) Selection of the optimal penalty parameter for LASSO regression. (c) LASSO–Cox regression analysis.

We performed univariate Cox regression analysis on the 145 OS-related differentially expressed ARGs and the hazard ratio (HR) value was calculated. With *P*  < 0.01 as the selection criterion, a total of 12 differentially expressed ARGs were obtained (Figure 3(a)). The 12 differentially expressed ARGs identified in this study may bear significance in relation to the prognostic implications of OS. It is anticipated that these ARGs may serve as potential markers guiding the clinical prognosis of patients afflicted with OS. According to the optimal penalty parameter (*λ*) value, the LASSO regression analysis determined that the optimal number of differentially ARGs associated with OS prognosis participating in the model construction was 11 (Figures 3(b) and 3(c)) [18]. The riskScore for each sample was calculated according to the prognostic model formula. The training cohort was divided into high-risk group (*N* = 43) and low-risk group (*N* = 43) according to the median riskScore. The validation cohort was divided into a high-risk group (*N* = 27) and a low-risk group (*N* = 26) according to the median riskScore.

### 3.4. Validation of the Risk Prognostic Model

**Figure 4:**
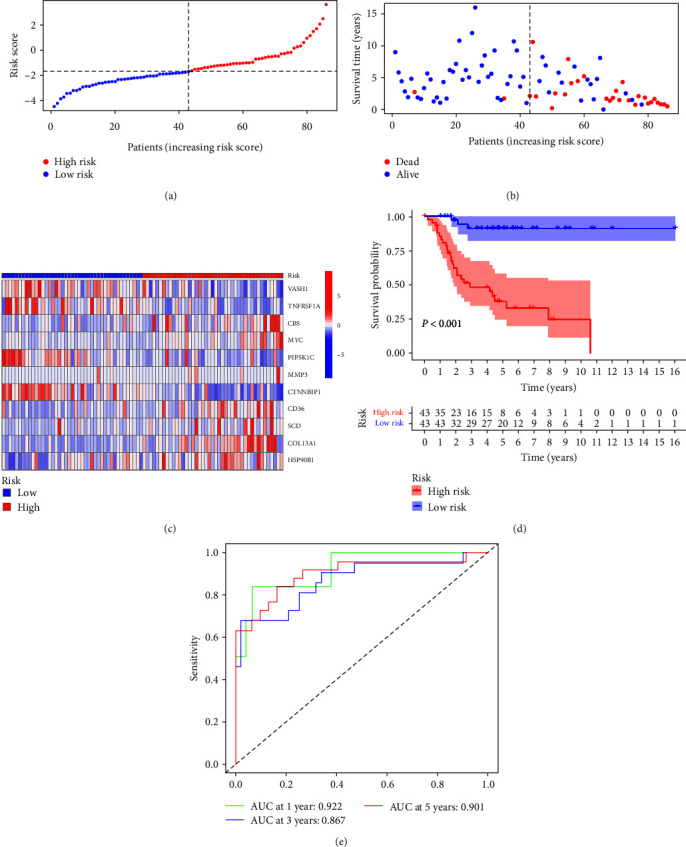
Training cohort: (a) risk curve, (b) survival status map, (c) risk heatmap, (d) survival curve, and (e) receiver operating characteristic curve.

**Figure 5:**
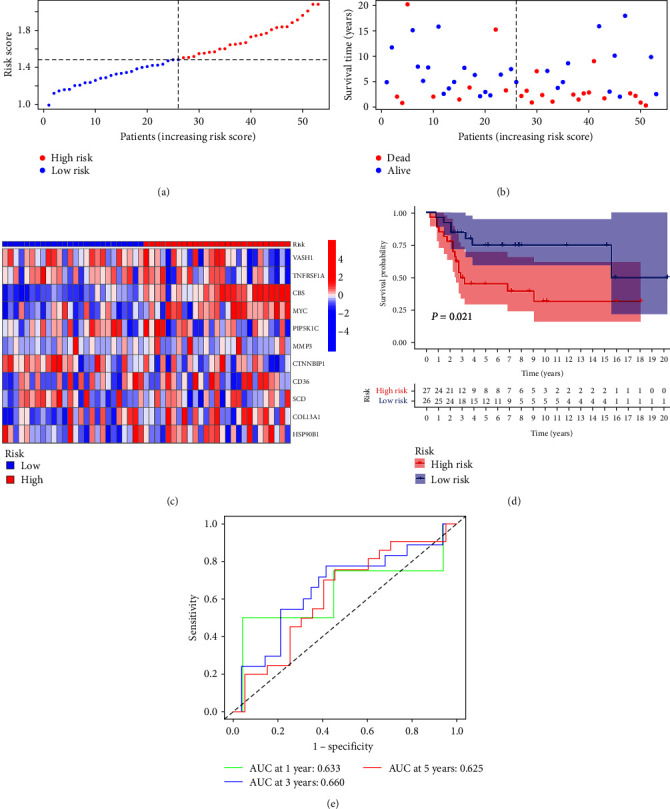
Validation cohort: (a) risk curve, (b) survival status map, (c) risk heatmap, (d) survival curve, and (e) receiver operating characteristic curve.

**Figure 6:**
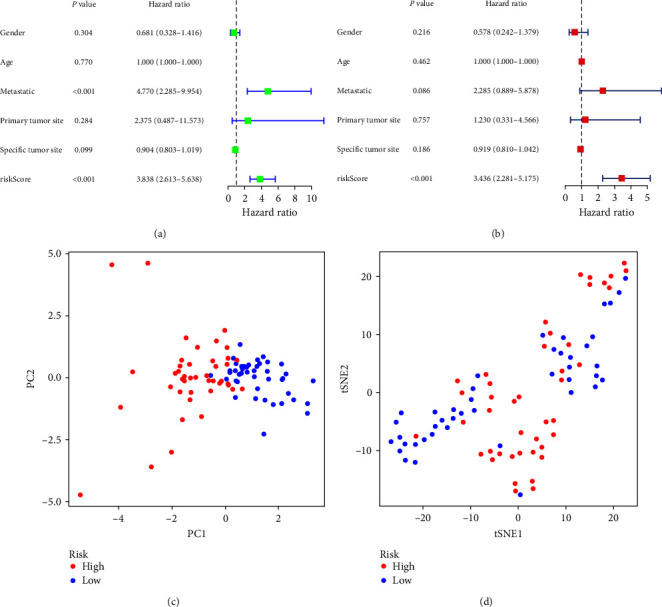
Training cohort: (a) univariate independent prognostic analysis, (b) multivariate independent prognostic analysis, (c) principal component analysis, and (d) t-distributed stochastic neighbor embedding analysis.

**Figure 7:**
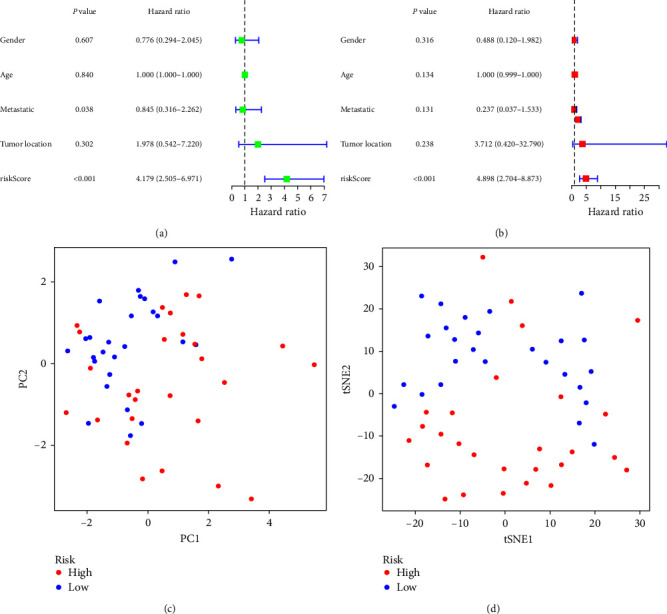
Validation cohort: (a) univariate independent prognostic analysis, (b) multivariate independent prognostic analysis, (c) principal component analysis, and (d) t-distributed stochastic neighbor embedding analysis.

The risk curve of the training cohort and validation cohort all show that from the low-risk group to the high-risk group, the risk of OS patients gradually increased (Figures 4(a) and 5(a)). The survival status map of the training cohort and validation cohort all show that the mortality in patients with OS increases with risk (Figures 4(b) and 5(b)). The risk heatmaps of the training cohort and validation cohort all show that from the low-risk group to the high-risk group, the expression levels of CBS, MYC, MMP3, CD36, SCD, COL13A1, and HSP90B1 gradually increase, which are high-risk ARGs, and the expression levels of VASH1, TNFRSF1A, PIP5K1C, and CTNNBIP1 gradually decrease, which are low-risk ARGs (Figures 4(c) and 5(c)). The survival curves of the training cohort and validation cohort all show that there was a difference in the survival of OS patients between the high- and low-risk groups (Figures 4(d) and 5(d)). The ROC curves of the training cohort and validation cohort all show higher areas under the ROC curve at 1, 3, and 5 years (Figures 4(e) and 5(e)). Univariate independent prognostic analysis of the training cohort and validation cohort all show that both riskScore and tumor metastasis can be used as independent prognostic factors (Figures 6(a) and 7(a)). Multivariate independent prognostic analysis of the training cohort and validation cohort all show that riskScore can be used as independent prognostic factors (Figures 6(b) and 7(b)).

### 3.5. PCA and t-SNE Analysis

PCA and t-SNE analyses were conducted on the risk prognostic models of the respective training and validation cohorts. Our findings revealed that the expression of the differentially ARGs associated with OS prognosis involved in the model construction significantly stratified patients into distinct high- and low-risk groups. This discernment strongly supports the model's accuracy (Figures 6(c), 6(d), 7(c), and 7(d).

### 3.6. Establish and Evaluate ARGs-Clinical Nomogram in the Training Cohort

**Figure 8:**
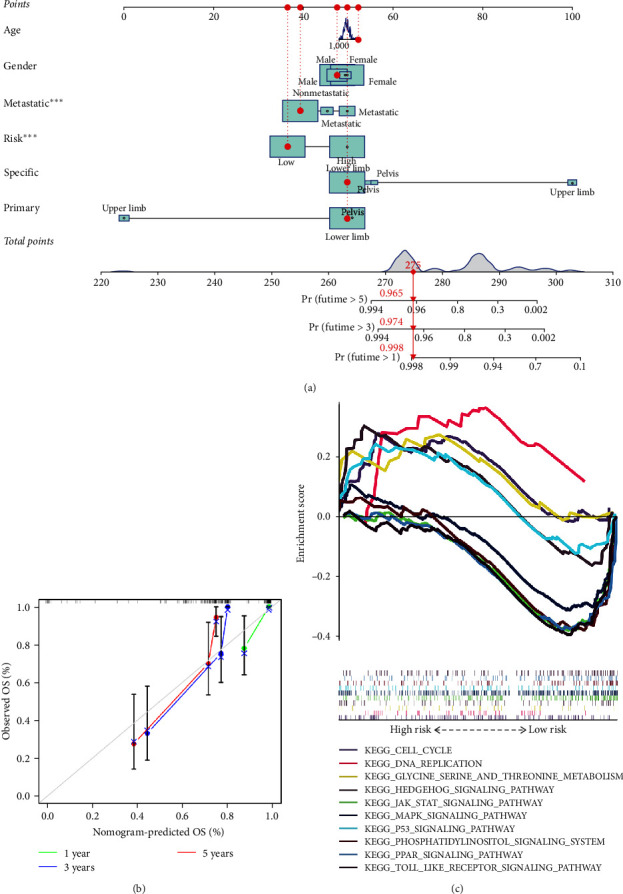
Training cohort: (a) nomogram, (b) calibration curve, and (c) gene set enrichment analysis. *⁣*^*∗∗∗*^*P* < 0.001.

The nomogram model was established based on riskScore and clinical features, to predict the 1-, 3- and 5-year survival rates of OS patients (Figure 8(a)). To determine the 1-, 3-, and 5-year survival rates for OS patients, six lines were drawn upward to measure the points of each factor in the nomogram. Subsequently, we plotted a line downward from the total points, which represented the sum of all points obtained from the upward lines. By intersecting this line with the survival axis, we were able to obtain the corresponding survival rates for the specified time intervals [23]. The calibration curve was close to the ideal curve (gray straight line), which suggested high consistency between the predicted result and actual result (Figure 8(b)). It suggestive that the better performance of the nomogram model in the prognostic prediction of OS patients.

### 3.7. Gene Set Enrichment Analysis

GSEA enrichment results showed that the active pathways in the high-risk group of the training cohort were cell cycle, DNA replication, glycine serine and threonine metabolism, Hedgehog signaling pathway, and P53 signaling pathway. Active pathways in the low-risk group of the training cohort were JAK-Stat signaling pathway, MAPK signaling pathway, phosphatidylinositol signaling system, PPAR signaling pathway, and Toll-like receptor (TLR) signaling pathway (Figure 8(c)).

### 3.8. Tumor Microenvironment Analysis

**Figure 9:**
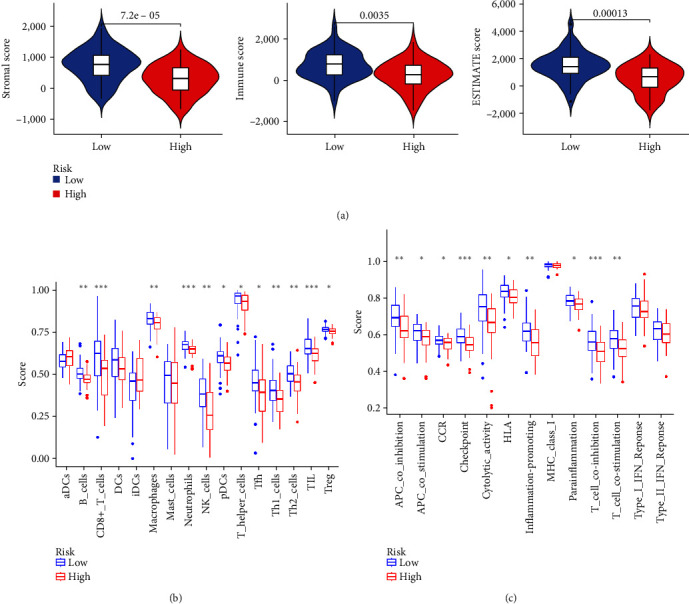
Training cohort: (a) differential analysis of tumor microenvironment; (b) immune cell difference analysis of single sample gene set enrichment analysis; and (c) immune function difference analysis of single sample gene set enrichment analysis. *⁣*^*∗*^*P* < 0.05, *⁣*^*∗∗*^*P* < 0.01, *⁣*^*∗∗∗*^*P* < 0.001.

The examination of the tumor microenvironment within the risk prognosis model pertaining to the training cohort revealed significant differences in stromal score, immune score, and overall score between groups categorized as high- and low-risk. Specifically, the scores observed within the low-risk group surpassed those identified in the high-risk group (Figure 9(a)).

### 3.9. Single-Sample Gene Set Enrichment Analysis

Differential analysis of immune cells in the risk prognostic model of the training cohort showed that CD8+_T_cells, neutrophils, and tumor-infiltrating lymphocytes (TIL) were significantly downregulated in the high-risk group (*P*  < 0.001; Figure 9(b)). Differential analysis of immune function showed that checkpoint and T_cell_coinhibition were significantly downregulated in the high-risk group (*P*  < 0.001; Figure 9(c)).

### 3.10. Immune Infiltration Cell Correlation Analysis

**Figure 10:**
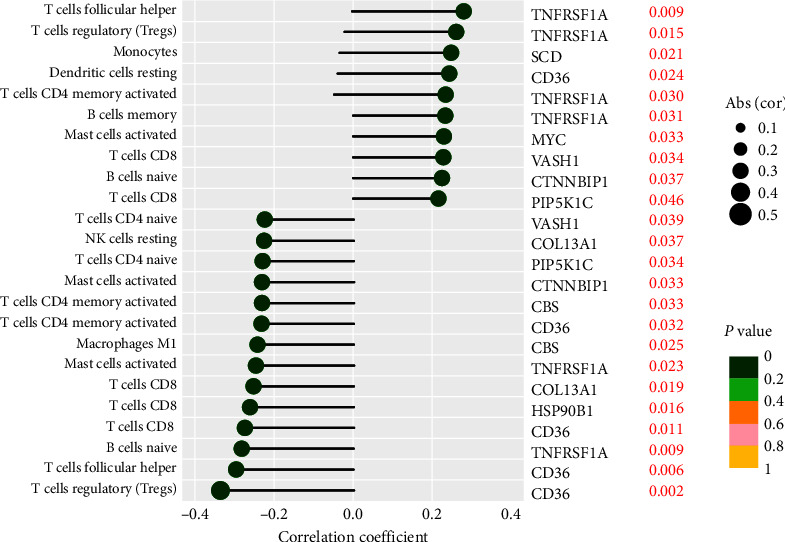
Correlation analysis between immune infiltration cells and anoikis-related genes involved in model construction.

**Figure 11:**
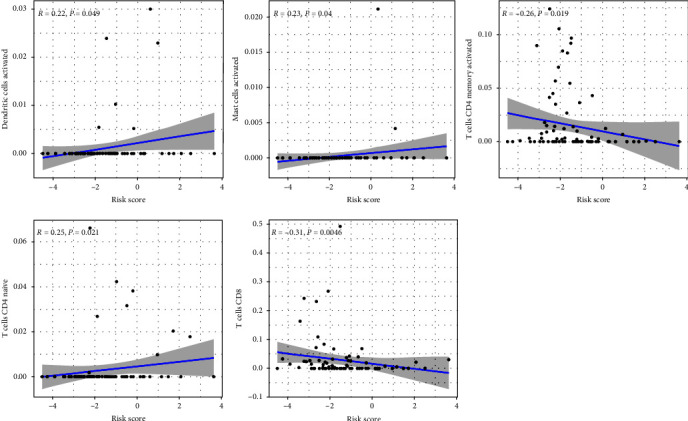
Correlation analysis between immune infiltration cells and riskScore of training cohort.

The immune correlation analysis showed that VASH1 was positively correlated with T cells CD8, and negatively correlated with T cells CD4 naive. TNFRSF1A was positively correlated with B cells memory, T cells CD4 memory activated, T cells follicular helper and T cells regulatory (Tregs), and negatively correlated with B cells naïve and Mast cells activated. CBS was negatively correlated with T cells CD4 memory activated and Macrophages M1. MYC was positively correlated with mast cells activated. PIP5K1C was positively correlated with T cells CD8 and negatively correlated with T cells CD4 naive. CTNNBIP1 was positively correlated with B cells naïve and negatively correlated with Mast cells activated. CD36 was positively correlated with dendritic cells resting, and negatively correlated with T cells CD8, T cells CD4 memory activated, T cells follicular helper, and Tregs. COL13A1 was negatively correlated with T cells CD8 and NK cells resting. HSP90B1 was negatively correlated with T cells CD8 (Figure 10). In addition, dendritic cells activated, mast cells activated, and T cells CD4 naive were positively correlated with riskScore of the risk prognostic model of the training cohort. T cells CD8 and T cells CD4 memory activated was negatively correlated with riskScore of the risk prognostic model of the training cohort (Figure 11). The positive correlation indicates that the higher the content of immune cells, the higher the risk of OS patients. The negative correlation indicates that the higher the content of immune cells, the lower the risk of OS patients.

### 3.11. Correlation Analysis between Clinical Features, ImmuneScore, and RiskScore

**Figure 12:**
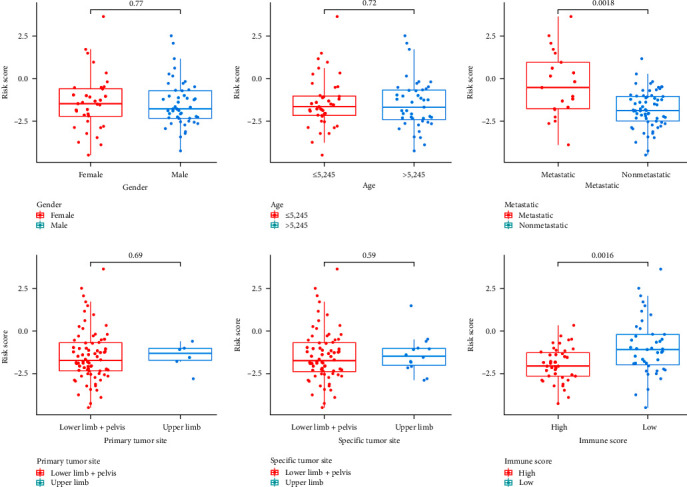
The differential analysis between riskScores of training cohort and different clinical features and immuneScores.

Correlation analysis between clinical features and riskScore indicates that the riskScore of the training cohort was different in clinical metastatic groups. Patients with metastatic OS are at higher risk than those nometastatic OS. Correlation analysis between immuneScore and riskScore indicates that the riskScore of the training cohort was different in high- and low-immuneScore groups. Patients with low immuneScore OS are at higher risk than those high immuneScore OS (Figure 12).

### 3.12. Differential Analysis of Immune Checkpoints

**Figure 13:**
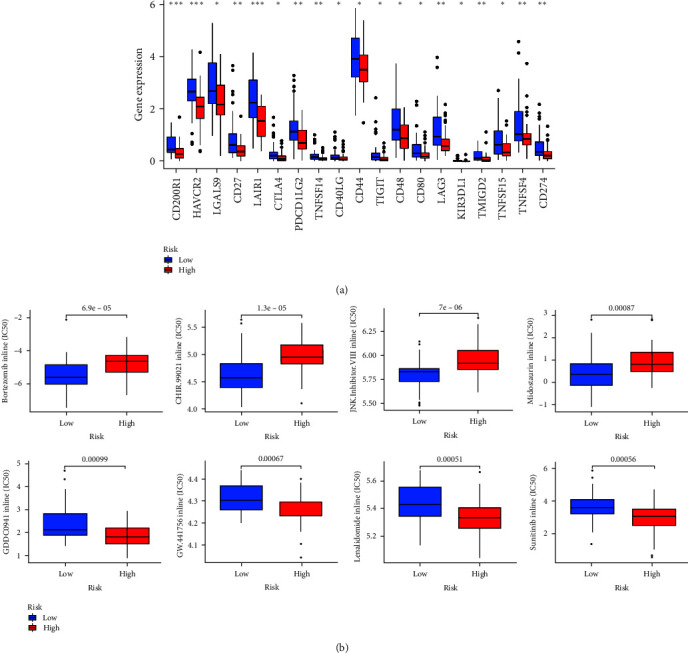
Training cohort: (a) immune checkpoint analysis and (b) drug sensitivity analysis. *⁣*^*∗*^*P* < 0.05, *⁣*^*∗∗*^*P* < 0.01, and *⁣*^*∗∗∗*^*P* < 0.001.

An examination of immune checkpoints indicated a noteworthy distinction of 19 genes associated with immune checkpoints between high- and low-risk groups within the training cohort. Particularly noteworthy were the genes CD200R1, HAVCR2, and LAIR1, demonstrating a remarkably high level of statistical significance (*P*  < 0.001; Figure 13(a)).

### 3.13. Drug Sensitivity Analysis

The drug sensitivity analysis of the risk prognostic model of the training cohort found that Bortezomib, Lenalidomide, Midostaurin Sunitinib, CHIR.99021, GDC0941, GW.441756, and JNK.Inhibitor.VIII had significant sensitivity in high- and low-risk groups (*P*  < 0.001). Patients in the low-risk group were more sensitive to Bortezomib, Midostaurin, CHIR.99021, and JNK.Inhibitor.VIII, and patients in the high-risk group were more sensitive to Lenalidomide, Sunitinib, GDC0941, and GW.441756 (Figure 13(b)).

### 3.14. Validation of ARGs Expression in OS

**Figure 14:**
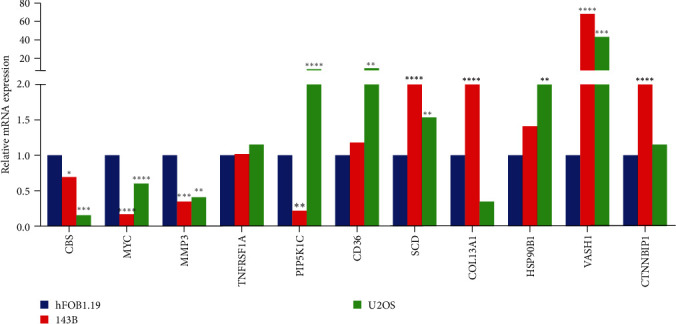
Validation of the mRNA expression level of ARGs in OS cell lines. Two OS cell lines (143B and U2OS) were selected for the assessment of their mRNA expression levels. The control group consisted of the normal osteoblast cell line hFOB1.19. *⁣*^*∗*^*P*  < 0.05, *⁣*^*∗∗*^*P*  < 0.01, *⁣*^*∗∗∗*^*P*  < 0.001, and *⁣*^*∗∗∗∗*^*P*  < 0.0001, each experiment was repeated three times.

Bioinformatic analysis revealed that in the construction of the risk prognosis model, 11 ARGs exhibited distinct expression profiles. Specifically, CBS, MYC, MMP3, TNFRSF1A, and PIP5K1C manifested low expression levels in OS, whereas CD36, SCD, COL13A1, HSP90B1, VASH1, and CTNNBIP1 demonstrated elevated expression levels in OS (Figure 1(a)). The details of primer pairs specific to the 11 AGRs and internal reference (GAPDH) are outlined in Table 1. To comprehensively evaluate the expression of ARGs, two OS cell lines were selected for the assessment of their mRNA expression levels. The control group consisted of the normal osteoblast cell line hFOB1.19. Comparative analysis revealed a significant downregulation of CBS, MYC, and MMP3 mRNA expression levels in both 143B and U2OS cell lines when compared to the normal osteoblast hFOB1.19. Furthermore, the mRNA expression level of SCD and VASH1 exhibited a significant increase in both OS cell lines (143B and U2OS) in comparison to hFOB1.19. In the 143B cell line, the mRNA expression level of PIP5K1C decreased compared to normal osteoblast hFOB1.19, while in the U2OS cell line, it increased. Additionally, the mRNA expression levels of COL13A1 and CTNNBIP1 were upregulated in the 143B cell line compared to the normal osteoblast hFOB1.19. Furthermore, the mRNA expression levels of CD36 and HSP90B1 were upregulated in the U2OS cell line compared to the normal osteoblast hFOB1.19 (Figure 14). The RT-qPCR outcomes for 10 out of the 11 analyzed ARGs aligned consistently with the findings derived from bioinformatics analysis. Specifically, the transcript levels of CBS, MYC, MMP3, and PIP5K1C exhibited a diminished expression profile in the context of OS. Conversely, the expression levels of CD36, SCD, COL13A1, HSP90B1, VASH1, and CTNNBIP1 were observed to be elevated in OS.

## 4. Discussion

In preceding investigations, several prognostic models were developed via bioinformatics analyses to investigate genes or lncRNAs associated with OS prognosis. The bioinformatics exploration of OS predominantly centers on the anticipation of OS-related genes. Distinguishing itself from prior research endeavors, this study innovatively incorporates the anoikis phenotype. The genes identified through the establishment of the prognostic model in this investigation exclusively consist of ARGs, which may actively participate in the progression of OS through the phenomenon of anoikis. Within the context of this study, an innovative prognostic model was formulated to systematically examine the prognostic outcomes of patients with OS, with a specific emphasis on the phenomenon of anoikis. The findings of this research contribute to the body of knowledge pertaining to OS prognosis. Furthermore, a more in-depth examination of the ARGs encompassed within the model holds the potential to significantly enhance clinical decision-making for patients afflicted with OS.

In this investigation, a training cohort was established utilizing the TCGA database, complemented by a validation cohort sourced from the GEO database. Through a comprehensive analysis of both repositories, a pioneering prognostic model predicated on ARGs was formulated. This model demonstrated notable efficacy in forecasting the survival prospects of patients diagnosed with OS, while concurrently offering insights into modulating the immune microenvironment among OS patients. Notably, the developed prognostic model exhibited the capability to forecast metastatic occurrences in OS patients. The construction of the model relied on the integration of 11 ARGs, among which CBS, MYC, MMP3, CD36, SCD, COL13A1, and HSP90B1 were classified as high-risk ARGs, whereas VASH1, TNFRSF1A, PIP5K1C, and CTNNBIP1 were categorized as low-risk ARGs. Patients grouped within the high-risk category demonstrated diminished immune scores encompassing immune cell populations and immune functionality compared to their low-risk counterparts. Specifically, a significant downregulation of CD8+ T cells, neutrophils, and TIL was observed within the high-risk cohort, along with a decrease in checkpoint molecules and T cell coinhibition markers. Furthermore, the authors identified 19 genes linked to immune checkpoints, such as CD200R1, HAVCR2, and LAIR1, which exhibited decreased expression levels in high-risk OS patients. Additionally, a nomogram model was developed by the authors, proving effective in prognosticating the outcomes for OS patients. Lastly, leveraging the risk prognostic model, the authors identified eight pharmaceutical agents displaying sensitivity. Bortezomib, Midostaurin, CHIR.99021, and JNK.Inhibitor.VIII were found to be more effective in low-risk OS patients, whereas Lenalidomide, Sunitinib, GDC0941, and GW.441756 exhibited greater sensitivity in high-risk OS patients. RT-qPCR was employed to authenticate 11 ARGs within the risk prognosis model. The findings elucidated that CBS, MYC, MMP3, and PIP5K1C exhibited diminished expression levels in OS, while CD36, SCD, COL13A1, HSP90B1, VASH1, and CTNNBIP1 manifested heightened expression levels in the context of OS.

The protein product derived from the CBS gene functions as a homotetramer, facilitating the enzymatic conversion of homocysteine to cystathionine—a pivotal initial step in the transsulfuration pathway. Allosteric activation of the encoded protein is achieved through adenosyl-methionine, with pyridoxal phosphate serving as a requisite cofactor. Genetic aberrations within this gene have been implicated in the manifestation of cystathionine beta-synthase deficiency (CBSD), a pathological condition associated with homocystinuria. The CBS1 domains, also referred to as CBS motifs, exert regulatory control over the functionality of numerous proteins across a spectrum of organisms, spanning from bacteria to humans. Notably, the CBS domain serves not only as a discerning energy sensor, modulating cellular activities in response to fluctuations in environmental cues but also as a regulator of intracellular chloride pathways, nitrate transit, and pyrophosphatase behavior [24]. CBS has also shown promise in predicting OS prognosis. Investigations suggest a downregulation of CBS in OS [25]. Moreover, recognized as both ferroptosis-related and immunogenic cell death-related genes, CBS not only forecasts OS patient survival but also significantly influences tumor chemoresistance [26–28]. This current study posits CBS as an ARG for appraising the prognosis of OS patients, further substantiating its prognostic relevance within this context. The MYC gene represents a proto-oncogene, characterized by its encoding of a nuclear phosphoprotein pivotal to the regulation of cell cycle progression, apoptosis, and cellular transformation. The resultant protein establishes a heterodimeric association with the cognate transcription factor MAX. Notably, amplification of this gene is frequently observed in various human malignancies. Diverse cellular processes such as cell development, cell cycle modulation, differentiation, apoptosis, angiogenesis, metabolism, DNA repair, protein translation, immune response, and stem cell formation are intrinsically influenced by MYC, primarily operating as a transcriptional regulatory factor [29]. Targeting MYC presents a promising avenue in cancer therapeutics owing to its ubiquitous dysregulation as a driver gene in human cancers [29]. Pertinently, studies elucidate that MYC inhibition reshapes the tumor immune microenvironment in OS by recruiting T lymphocytes and engaging the CD40/CD40L system [30]. Additionally, the interplay between miR-193b and MYC has been associated with decelerating the progression and metastasis of OS [31]. Noteworthy potential biomarkers for forecasting the trajectory and prognosis of OS, thereby improving clinical therapeutic strategies, encompass the evaluation of c-MYC protein expression and the apoptosis index [32]. This reinforces the close linkage of MYC with OS and underscores its predictive value, further corroborating the findings of this study. The members of the MMP family play integral roles in the degradation of the ECM during various physiological phenomena, including embryonic development, reproductive processes, and tissue remodeling. Additionally, these enzymes are implicated in pathological conditions such as arthritis and metastasis. The majority of MMPs are initially secreted as inactive proproteins, a state from which they undergo activation through cleavage by extracellular proteinases. Extracellular vesicles play a pivotal role in facilitating the molecular transfer of MMP3, thereby augmenting tumor proliferation and onset. This underscores MMP3 as a multifaceted determinant crucial for tumor progression [33]. Combining MMP3 therapy with oncolytic virus therapy presents a promising avenue in cancer therapeutics [34]. Studies underscore miR-134′s capacity to target MMP1 and MMP3, effectively impeding OS cell invasion and metastasis both in vitro and in vivo [35]. Additionally, compounds like the curcumin analog DK1 can suppress prometastatic genes and proteins including MMP3, thereby reducing the metastatic and angiogenic potential of OS cell lines [36]. Notably, MMP3 has been implicated in stimulating tumor cell metastasis in OS [36]. This study identifies MMP3 as a high-risk actionable predictive gene in OS, indicating its role as an independent prognostic factor in our risk prognostic model. These findings align coherently with earlier investigations, affirming the consistency of the results presented herein.

The protein encoded by CD36 constitutes the fourth principal glycoprotein situated on the surface of platelets, functioning as a receptor for thrombospondin in both platelets and diverse cellular lineages. Given the ubiquitous presence of thrombospondins, proteins with broad distribution participating in numerous adhesive processes, it is plausible that this particular protein assumes pivotal roles as a cell adhesion molecule. The transmembrane glycoprotein CD36 serves as a crucial regulator influencing various cellular processes such as apoptosis, immune detection, inflammation, and lipid uptake. Its involvement in reprograming lipid metabolism and modulating tumor-associated immune cells has been associated with cancer growth and the establishment of tumor immune tolerance [37]. The potential targeting of CD36 in cancer therapy has been proposed due to these multifaceted roles. Studies have indicated a correlation between CD36 and OS progression. Specifically, downregulation of the thrombospondin receptor CD36 by ribozymes has been observed to decelerate the development of the human OS cell line [38]. However, uncertainties persist regarding the specific contribution of CD36 to OS evolution. Our research indicates that CD36, identified as a high-risk prognostic gene, not only serves as a predictor of OS survival but also exerts influence over the immune microenvironment within OS. SCD gene encodes an enzymatic entity intricately implicated in the intricate processes of fatty acid biosynthesis, with a principal focus on the synthesis of oleic acid. The encoded protein is a constituent of the fatty acid desaturase family and assumes the characteristic feature of an integral membrane protein, spatially localized within the endoplasmic reticulum. Notably, the enzymatic production of monounsaturated fatty acids by SCD has been linked to critical cellular processes encompassing cell development, survival, differentiation, metabolic regulation, and signaling. In the context of cancer, SCD contributes to metabolic reprograming along canonical Wnt signaling pathways and activation of YAP, thereby promoting stemness and tumorigenesis [39]. Consequently, the role of SCD as a potential therapeutic target in cancer treatment has gained attention due to its pivotal involvement in tumor lipid metabolism and membrane architecture [40]. Furthermore, upregulation of SCD-1 has been identified as exerting a self-protective impact in MG63 OS cells following high shear force injury [41]. Our analysis underscores the prognostic value of CD36 and SCD in predicting OS patient survival, opening new avenues for research into enhancing OS prognosis. The COL13A1 gene encodes an alpha chain of nonfibrillar collagens, the precise functional attributes of which remain enigmatic. Nevertheless, its discernible expression within cells responsible for the synthesis of connective tissue, albeit at minimal levels, intimates a conceivable overarching involvement in the regulation of connective tissues. Earlier research has identified COL13A1 as a prognostic gene in OS, influencing patient survival through immune-related multiomics studies [42]. COL13A1 emerges as a predictive marker for OS patient outcomes, impacting pyroptosis prognoses and DNA methylation control [43, 44]. This study reaffirms the prognostic significance of COL13A1 as an ARGs for OS, shedding light on its pivotal role in predicting OS patient outcomes. HSP90B1 pertains to a constituent within the adenosine triphosphate (ATP)-metabolizing molecular chaperone family, exhibiting pivotal functions in the stabilization and folding of diverse proteins. The specified protein is predominantly situated in melanosomes and the endoplasmic reticulum. Notably, the expression of this protein is implicated in various pathogenic conditions, including the initiation of tumorigenesis. HSP90B1, known as gp96, functions as a chaperone for TLRs and is expressed in T-cells, primarily localizing to the endoplasmic reticulum [45]. Despite its previous association with OS survival [46], the precise mechanistic connection between HSP90B1 and OS survival remains ambiguous. This study delineates HSP90B1 as a high-risk gene associated with OS development, with increased expression correlating with an unfavorable prognosis for OS patients. The insights from this investigation significantly advance our understanding of OS survival dynamics, emphasizing the critical role of HSP90B1 therein.

The VASH1 protein demonstrates the capacity for actin-binding activity and metallocarboxypeptidase activity. It plays a pivotal role in the negative regulation of angiogenesis, suppression of blood vessel endothelial cell migration, and mediation of proteolytic processes. Activated vascular endothelial cells demonstrate a heightened preference for expressing VASH1, an emerging endogenous modulator of angiogenesis. Numerous malignancies, such as upper urinary tract urothelial carcinoma, ovarian carcinoma, squamous cell carcinoma of the esophagus, renal cell carcinoma, and breast carcinoma, exhibit significant VASH1 expression based on several studies [47]. Nonetheless, scant investigations have explored VASH1's correlation with OS, with limited reports indicating its role in obstructing adriamycin resistance in OS cells through activation of the protein kinase B signaling pathway [48]. This study unravels the prognostic influence of VASH1, categorized as a low-risk ARG, on the survival of OS patients. As such, this work heralds a novel avenue for delving into the prognosis and clinical management of OS. The TNFRSF1A gene serves as the encoding locus for a constituent of the tumor necrosis factor (TNF) receptor superfamily. The ensuing receptor experiences proteolytic cleavage, thereby releasing its soluble variant capable of interacting with unbound TNF-*α*, thereby mitigating inflammatory responses. This interaction assumes significance in cellular processes such as survival, apoptosis, and inflammation, as elucidated in prior investigations [49]. Earlier research has underscored the prognostic relevance of the pyroptosis-related gene TNFRSF1A concerning OS patients' survival [43]. This investigation showcases TNFRSF1A's potential as an ARG for predicting the prognosis of OS patients, emphasizing its utility in forecasting their survival. CTNNBIP1 is the gene responsible for encoding a protein that interacts with *β*-catenin, thereby operating as a negative regulator within the intricate Wnt signaling pathway. The precise classification of its role as either a tumor suppressor or an oncogene in various cancer types is currently a matter of considerable debate in the scientific community [50]. Studies have highlighted CTNNBIP1's influence on OS patient survival by functioning as both a ferroptosis- and pyroptosis-related gene [51, 52]. This study identifies CTNNBIP1 as a low-risk gene when analyzing OS patient survival as an ARG, opening avenues for expanded research in this domain. PIP5K1C encodes a type I phosphatidylinositol 4-phosphate 5-kinase, a pivotal enzyme instrumental in the phosphorylation of phosphatidylinositol 4-phosphate, thereby generating phosphatidylinositol 4,5-bisphosphate. This enzymatic entity is prominently localized at synapses and has been discerned to actively participate in cellular processes such as endocytosis and cell migration. Notably, mutations occurring at this specific genomic locus have been causally linked to the manifestation of lethal congenital contractural syndrome. The association between PIP5K1C and OS lacks conclusive evidence. To the best of my knowledge, this study marks the initial identification of PIP5K1C's impact on the immunological microenvironment and prognosis of OS patients. This newfound discovery advocates for a fresh inquiry aimed at enhancing the clinical effectiveness and prognostication of OS.

The depletion of T cells constitutes a pivotal element in OS pathophysiology [53]. Research findings indicate that individuals with heightened OS risk factors exhibit diminished overall counts of CD8+ T cells. Furthermore, investigations revealed the involvement of C3 TXNIP+ and C5 IFIT1+ macrophages in the regulation of regulatory T cells and their participation in the exhaustion of CD8+ T cells. This underscores the potential of immunotherapeutic interventions capable of targeting both CD8+ T cells and macrophages [53]. A comprehensive study on the hypoxic-related prognosis model of OS demonstrated the downregulation of five distinct immune cell types—DCs, macrophages, neutrophils, pDCs, and TIL—as well as three distinct immunological functions—CCR, APC coinhibition, and checkpoint signaling—in the high-risk group [54]. Moreover, the ARGs risk prognosis model developed in this research indicated reduced expression of neutrophils, TIL, and checkpoint genes in the high-risk group of OS patients. Contrasting metastatic OS with nonmetastatic OS, the latter exhibited higher quantities of CD56 bright natural killer cells, immature B cells, M1 macrophages, and neutrophils, while the former demonstrated lower levels of M2 macrophages [55]. The study identified tumor metastasis as an independent prognostic factor in the ARGs-based risk prognosis model and noted reduced neutrophil expression in the high-risk group and patients with metastatic OS. TIL have been implicated in the progression of various malignancies. Moreover, TIL therapy, having shown success in treating multiple malignancies such as melanoma, may emerge as a potentially effective approach for adult OS treatment [56]. The expression of T cell coinhibition-related genes is notably diminished in the high-risk group, a characteristic evident in the zinc finger protein gene-based prognostic marker for OS, further supported by a cuproptosis-related lncRNAs-based OS risk prognostic model [18, 57].

In our analysis focusing on the prognostic factors in OS, our ARGs risk prognosis model revealed significantly diminished expression of three immune checkpoint-related genes: CD200R1, HAVCR2, and LAIR1 in the high-risk group. Existing literature suggests that HAVCR2 serves as an immunological marker for OS and holds potential significance in determining patient prognosis [51]. Furthermore, elevated expression of HAVCR2, an immune checkpoint, is associated with better OS prognosis [58]. Utilizing pyroptosis-related lncRNAs, a risk-predictive model for OS underscored the underexpression of LAIR1 within high-risk populations [59]. Notably, in OS cells, overexpression of LAIR1 led to decreased expression of glucose transporter-1 and hindered the production of components linked to epithelial-mesenchymal transition [60]. Conversely, there remains an absence of reported correlation between CD200R1 and OS. Our study unveiled decreased expression of the immune checkpoint-related gene CD200R1 in the high-risk group identified through the ARGs-based OS risk prognosis model. This novel discovery introduces a promising avenue for further investigations into OS survival research, potentially contributing to advancements in OS immunotherapy strategies. Moreover, it is imperative to direct attention toward immunoactivation or inhibition markers such as CTLA4 and LAG3. Immunotherapy has emerged as a promising therapeutic strategy for the treatment of human malignancies. The evasion of immune surveillance is widely acknowledged as a significant contributor to malignant progression. Notably, suppressor receptors, particularly CTLA4 and PD1, play a pivotal role in mediating antitumor immune effects [61]. Evidence reveals an augmented expression of CTLA4 and an increased proportion of monocytes in patients diagnosed with OS [62]. The polymorphism of the CTLA4 gene may exhibit an association with the risk of OS in the Chinese Han population, thereby serving as a molecular marker for assessing the OS risk [63]. In patients with OS, particularly those with metastatic or pathological fractures, there is a noteworthy upregulation of PD1 expression in T cells [64]. The concurrent blockade of CTLA4 and PD1 emerges as an attractive immunotherapeutic approach for patients grappling with drug-resistant or metastatic OS [61]. Additionally, a compelling finding suggests that the triple blockade of CTLA4, PDL1, and TIM3 represents an effective strategy for inhibiting the progression and migration of tumor cells in OS [65].

The in vitro invasive capacity of OS cells is notably attenuated by bortezomib, an established anticancer medication categorized as a selective and reversible inhibitor of the ubiquitin-dependent proteasome system. This compound induces cancer cell cycle arrest and apoptosis [66]. Concurrent administration of kinase inhibitors such as Midostaurin with the cytokine oncostatin-M holds promise as novel adjunctive therapies for managing this highly aggressive cancer. Notably, oncostatin-M has exhibited the ability to sensitize rat OS cells to apoptosis/necrosis [67]. Recent research indicates that the cytotoxic effects of RG-7388 on SJSA-1 OS cells were counteracted by the GSK-3 inhibitor CHIR-99021 through the reduction of Bak protein levels [68]. These findings align with our previous research, suggesting that Lenalidomide holds sensitivity as a therapeutic agent for OS within the cuproptosis-related lncRNA risk prediction model [18]. Further investigations have revealed the therapeutic efficacy of sunitinib against OS. Utilizing redox-responsive zwitterionic hydrogels for local delivery of sunitinib and ce6 has proven effective in preventing OS recurrence [69]. Moreover, Sunitinib exhibits immunomodulatory effects in tumor-bearing animals by targeting STAT3 and inhibiting PD-L1 expression specifically in OS [70]. Despite these advancements, the association between JNK.Inhibitor. VIII, GDC0941, GW.441756, and OS remains inadequately documented. Nevertheless, findings from this study underscore the sensitivity of these medications for OS, suggesting their potential utility in augmenting the clinical efficacy of OS treatment in future research endeavors.

The present study is subject to certain limitations that warrant acknowledgment. First, the sample size of OS cases incorporated in this investigation is comparatively diminutive when juxtaposed with cohorts examining other neoplastic entities. This discrepancy can be attributed, in part, to the relatively low incidence of OS. It is anticipated that, with the ongoing expansion and refinement of the utilized database, future investigations will benefit from larger and more diverse OS cohorts. Second, elucidating the intricate biological functions of the identified ARGs is imperative for comprehending their capacity to prognosticate and modulate the immune microenvironment in the context of OS. The precise influence of these ARGs on the pathogenesis of OS necessitates further scrutiny through intensified in vivo and in vitro experimentation. Subsequent research endeavors should endeavor to delineate the nuanced roles played by these ARGs in the progressive trajectory of OS disease.

## 5. Conclusion

In this investigation, a novel prognostic model for OS has been successfully formulated by incorporating 11 distinct ARGs. The model exhibits robust predictive capabilities, particularly in the context of patient survival and the intricate immune microenvironment. Furthermore, a nomogram model has been developed, characterized by exceptional predictive accuracy in evaluating the prognosis of patients with OS in terms of overall survival. Additionally, the study has identified eight pharmacological agents with promising potential for efficacious intervention in the treatment of OS. These findings make a substantial contribution to the enhancement of clinical outcomes and survival rates among OS patients. The prognostic model established in this investigation carries significant implications for clinical practice, exerting a noteworthy impact on the prognosis of individuals afflicted with OS. Furthermore, it holds considerable promise in refining treatment strategies and elevating the standard of clinical care for OS patients.

## Figures and Tables

**Table 1:** Primer sequences for RT-qPCR.

Genes	Forward	Reverse
CBS	AGTTGGCAAAGTCATCTACAAGCA	AACACCATCTGCCGCTGACT
MYC	CCTGGTGCTCCATGAGGAGA	CAGTGGGCTGTGAGGAGGTTT
MMP3	GGACAAAGGATACAACAGGGAC	GCTTCAGTGTTGGCTGAGTG
TNFRSF1A	ACGAAGTTGTGCCTACCCC	GAGGGATAAAAGGCAAAGACCAA
PIP5K1C	GTTCAATCGCTCCGCCTGTC	GATTGTCACGCACCAGACCAC
CD36	AGCCACAAACCAAGAATCTACCTG	CTTCCCAGTTAAAAGGAAAGGCACT
SCD	GCTACACTTGGGAGCCCTGTATG	AGACGATGAGCTCCTGCTGTTATG
COL13A1	ATGGAAACATCAATGAGGCTCT	CCTTTTCCCCATCGTGTCCT
HSP90B1	TAATCCCAGACACCCGCTGA	ATACCCTGACCGAAGCGTTG
VASH1	GAAACATGGGTGTGCTTGGC	TCGGGGAAAGTGACAACAGG
CTNNBIP1	CAGCAGAGGCGTACTACCCA	CTCCCCTTTCCAAGATGACCC
GAPDH	GCACCGTCAAGGCTGAGAAC	TGGTGAAGACGCCAGTGGA

## Data Availability

The data supporting the results of the study are available from the TCGA database(https://portal.gdc.cancer.gov/) and GEO database (https://www.ncbi.nlm.nih.gov/geo/).
